# Breeding synchrony and predator specialization: A test of the predator swamping hypothesis in seabirds

**DOI:** 10.1002/ece3.4863

**Published:** 2019-01-15

**Authors:** Sébastien Descamps

**Affiliations:** ^1^ Norwegian Polar Institute, Fram Centre Tromsø Norway

**Keywords:** colonial seabird, functional response, hatching synchrony, stabilizing selection

## Abstract

Reproductive synchrony is a widespread phenomenon that is predicted to be adaptive for prey with specialist predators but not for those with generalist ones. I tested this prediction in three polar seabird species characterized by different levels of predator specialization. In the Antarctic petrel, for which the only predator was highly specialized, hatching dates were highly synchronous and chicks that hatched close to the mean hatching date had a higher survival. In black‐legged kittiwakes and Brünnich's guillemots, whose predators were generalists, breeding was less synchronous and there was no fitness advantage in hatching close to the mean. This study emphasizes the potential importance of the relative timing of reproduction for individual fitness and supports the hypothesis that the adaptive value of breeding synchrony depends on the predator functional response.

## 1 INTRODUCTION

Reproductive synchrony is a widespread phenomenon in both the plant and the animal kingdom, and several hypotheses have been proposed to explain its adaptive advantages (Ims, 1990a). One of these hypotheses proposes that breeding at the same time as other members of the population represents an antipredation strategy (Darling, 1938). Indeed, synchronous breeding may allow for a better collective defense against predators. In addition, the presence of a large number of offspring at the same time may decrease the predators’ ability to capture prey (predator confusion) and may swamp the predator population (Hatchwell, 1991; Ims, 1990a). This predator swamping hypothesis has often been proposed as the ultimate explanation for synchronous breeding in systems like colonial birds (e.g., Burr et al., 2016; Descamps, Forbes, Gilchrist, Love, & Bêty, 2011; Findlay & Cooke, 1982; Gochfeld, 1980; Hatchwell, 1991; Lepage, Gauthier, & Menu, 2000; Williams, 1975). However, the predator functional response is a key component to consider before assessing whether or not breeding synchrony is adaptive (Ims, 1990b). Indeed, Ims (1990b) demonstrated with the use of simulations that synchronous reproduction should be favored when facing predation from specialist predators, while asynchrony should be favored when the predators are generalists, especially when prey switching occurs at relatively high offspring densities. Testing this model in the wild is difficult, which is why the role of predator functional response has largely been overlooked in assessing the adaptive value of synchronous breeding.

Here, I used data on hatching date in three different species of colonial seabirds characterized by different levels of predator specialization to test the Ims model (Ims, 1990b) and the adaptive value of breeding synchrony as a function of predator specialization. The three species considered were colonial seabirds breeding at high latitudes in polar environments: the Antarctic petrel *Thalassoica antarctica* from the Svarthamaren colony (Dronning Maud Land, Antarctica), the black‐legged kittiwake *Rissa tridactyla,* and Brünnich's guillemot *Uria lomvia* from Svalbard (Grumantbyen and Ossian Sarsfjellet, respectively). Eggs and chicks from Antarctic petrels at Svarthamaren are virtually the only prey of the south polar skua *Stercorarius maccormicki* during the breeding season (Brooke, Keith, & Røv, 1999), which is de facto a highly specialized predator. In contrast, eggs and chicks from black‐legged kittiwakes and Brünnich's guillemots are preyed upon by Glaucous gulls *Larus hyperboreus *and Arctic foxes *Vulpes lagopus *that feed on a wide range of prey (Bustnes, Erikstad, Bakken, Mehlum, & Skaare, 2000; Eide, Jepsen, & Prestrud, 2004) and are therefore generalist predators. Thus, I tested the Ims model and the prediction that breeding close to the hatching peak should be of higher adaptive value (i.e., resulting in higher reproduction success) in the Antarctic petrel, compared to the black‐legged kittiwake and Brünnich's guillemot.

## 2 METHODS

### 2.1 Study systems

The study took place in Antarctica and Svalbard. In Antarctica, it was carried out during four breeding seasons (2011/12, 2012/13, 2013/14, and 2017/18) at the Svarthamaren Antarctic petrel colony (71°53’S, 5°10’E) in Dronning Maud Land, which holds between 100,000 and 200,000 breeding pairs and is located 200 km from the coast (Descamps et al., 2016). The Antarctic petrel is a medium‐sized petrel that weighs ca. 600 g and breeds on the ground in scree slopes. They lay a single egg at the end of November/early December, with both parents incubating and feeding the chick. Nests are densely located (0.8 breeding pairs per m^2^, Mehlum et al. 1988). The only predator at the Svarthamaren colony is the south polar skua, which preys almost exclusively on Antarctic petrel eggs and chicks (Brooke et al., 1999, pers. obs.).

In Svalbard, breeding phenology and chick survival data were collected in seven (2011–2017) and six (2012–2017) consecutive years for black‐legged kittiwakes and Brünnich's guillemots, respectively. Black‐legged kittiwake reproduction was monitored at the Grumantbyen colony in Isfjorden (78°17’N 15°10’E) and Brünnich's guillemot reproduction at the Ossian Sarsfjellet colony in Kongsfjorden (78°93’N 12°44’E). The Grumantbyen colony holds approximately 45 pairs of kittiwakes and the Ossian sarsfjellet 1,000 pairs of guillemots (as well as 2,000 pairs of kittiwakes that breed sympatrically with the guillemots). Black‐legged kittiwakes are colonial cliff breeders that typically lay one or two eggs in Svalbard (Strøm, 2006a). They feed mostly on fish, crustaceans, and other marine invertebrates (Vihtakari et al., 2018). Brünnich's guillemots are colonial cliff breeders and lay a single egg. Their diet consists mainly of fish and crustaceans (Anker‐Nilssen et al., 2000; Strøm, 2006b). In both species, females and males share the incubation and chick‐rearing duties (Coulson, 2011; Gaston & Jones, 1998). During the breeding season and in the study colonies, the only predators of Brünnich's guillemots were the glaucous gull and Arctic fox (pers. obs.), while the only predator of kittiwake chicks was the glaucous gull (kittiwake chicks at the Grumantbyen colony were inaccessible to foxes). Glaucous gulls and Arctic foxes are generalist predators and their diets consist of a wide variety of prey (Anker‐Nilssen et al., 2000; Bustnes et al., 2000; Eide et al., 2004; Varpe, 2010).

In all three study sites, nests were monitored every 2–5 days from early or mid‐incubation until the mid‐chick‐rearing period. Direct observations were used to determine the presence of egg(s) or chick(s) in each nest, and thus the hatching date and chick survival at the individual level. The annual number of nests monitored annually is presented in Table 1, and only nests where hatching date could be estimated with an accuracy of ±2 (petrels and kittiwakes) or ±3 (guillemots) days were included in the analyses. Due to logistical constraints, I could not monitor nest status until fledging but could only assess chick survival until the age of 20 days (Antarctic petrels) or 15 days (Brünnich's guillemots and black‐legged kittiwakes). For kittiwakes, I considered the probability that at least one chick survived up to 15 days. The guillemot nest monitoring thus stopped just before chicks began to jump from the cliff. This jumping event can sometimes be associated with high mortality but apparently only in colonies where chicks do not directly land in the water (Hatch, 1983; Williams, 1975). In colonies where chicks land directly in the sea (as in the study colony), chick predation rates associated with this jumping event are not higher (Gilchrist & Gaston, 1997; Williams, 1975).

**Table 1:** Hatching dates in Antarctic petrels (Svarthamaren, Dronning Maud Land), black‐legged kittiwakes (Grumantbyen, Svalbard), and Brünnich's guillemots (Ossian Sarsfjellet, Svalbard)

		Mean hatching date	Range	*n*
Antarctic petrel	2011	14 Jan	9–21 Jan	89
	2012	14 Jan	10–21 Jan	205
	2013	15 Jan	10–22 Jan	89
	2017	9 Jan	5–13 Jan	208
Black‐legged kittiwake	2011	9 Jul	6–14 Jul	19
	2012	7 Jul	2–15 Jul	31
	2014	13 Jul	10–19 Jul	30
	2015	10 Jul	4–19 Jul	15
	2016	7 Jul	1–21 Jul	37
	2017	13 Jul	8–20 Jul	25
Brünnich's guillemot	2012	7 Jul	2–23 jul	18
	2013	3 Jul	23 Jun−21 Jul	25
	2014	6 Jul	22 Jun−21 Jul	30
	2015	2 Jul	22 Jun−18 Jul	30
	2016	03 Jul	23 Jun−19 Jul	16
	2017	04 Jul	20 Jun−16 Jul	30

### 2.2 Statistical methods

To test for a relationship between hatching date and chick survival, I performed generalized mixed‐effect models with a logit link function and a binomial error distribution. Nest identity was included as a random factor to take into account pseudoreplication in the data (i.e., many nests were monitored in several seasons and were thus associated with several hatching dates and chick survival data). I used maximum likelihood (ML) to compare models with different fixed effects (but the same random factor). I considered either a linear or a quadratic effect of hatching date to test the predictions. All hatching dates were centered on their mean for each species and year, so that any interannual difference in mean hatching dates would not affect the results and conclusions. I used a model selection approach based on the Akaike's information criterion (Akaike, 1973; Burnham, 2002) to identify the model with the strongest support. All analyses were performed in the software R 3.2.4 (R Development Core Team, 2016) using the glmer function of package *lme4* (Bates, Maechler, & Walker, 2015).

## 3 RESULTS

**Figure 1:**
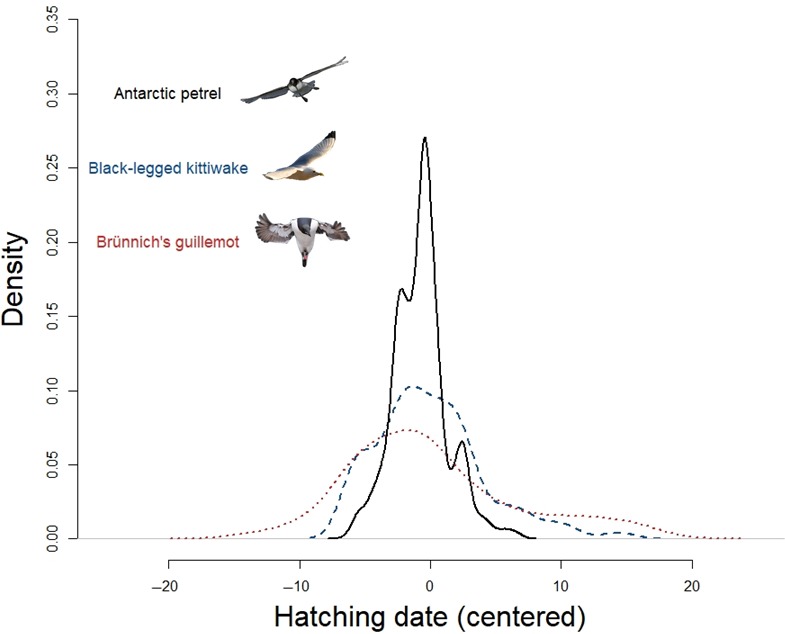
Density distributions of hatching dates in three seabird species. Solid black line represents hatching dates of Antarctic petrels at Svarthamaren, Dronning Maud Land, dashed blue line hatching dates of black‐legged kittiwakes at Grumantbyen, Svalbard and dotted red line hatching dates of Brünnich's guillemots at Ossian Sarsfjellet, Svalbard. Hatching dates are centered on their mean for each species and year. Density distributions are kernel density estimations calculated with the densityplot function from package lattice in R (Sarkar, 2008)

**Figure 2:**
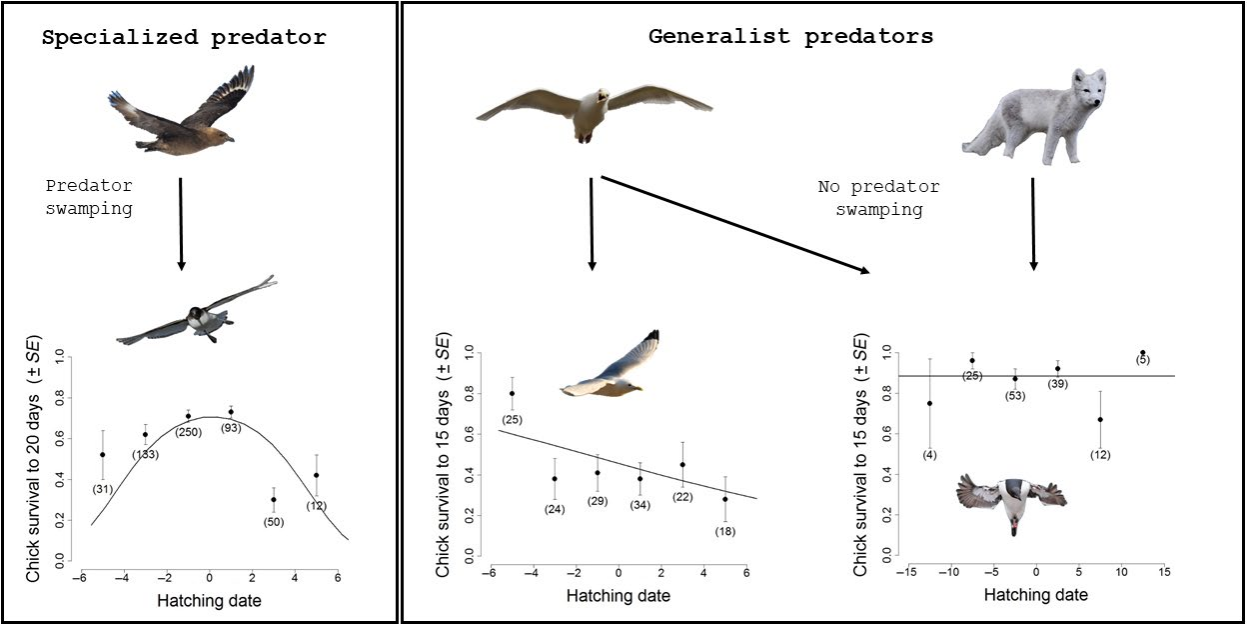
Chick survival probability as a function of hatching date in three seabird species. Left panel represents the probability to survive to 20 days after hatching for Antarctic petrel chicks; the south polar skua is the only predator and is highly specialized on Antarctic petrel chicks. The central and right panels represent the probability of having at least one chick/the chick surviving to 15 days after hatching for black‐legged kittiwake (center) and Brünnich's guillemot (right) chicks. The glaucous gull is one of the main predators of kittiwake and guillemot chicks but also feeds on many other sources. The Arctic fox is also a predator of guillemot chicks in the studied colony (Ossian Sarsfjellet, Svalbard) and also preys on many other sources. The lines in each panel represent the predicted average relationships between hatching dates (centered) and chick survival probability based on linear mixed models. These lines have been estimated using individual hatching date (continuous variable) and chick survival (binary variable) while the black symbols represent the observed survival probabilities (and their associated SE and sample size) for 2‐day hatching date intervals

The Antarctic petrel had a very high hatching synchrony (Figure 1), and 68% of the hatching occurred within 4 days (Table 1). In contrast, hatching dates of black‐legged kittiwakes and Brünnich's guillemots occurred during a two‐ to four‐week period, respectively (Table 1), and hatching was more evenly spread compared to petrels (Figure 1). Variance in petrel hatching date (all years combined and data centered on their mean for each year) was indeed significantly smaller than those for kittiwakes (*F*_153,590_ = 3.45, *p* < 0.001) and guillemots (*F*_139,590_ = 11.37, *p* < 0.001). Hatching close to the peak had significant fitness advantages for petrels but not for kittiwakes and guillemots (Figure 2). Survival was significantly higher for Antarctic petrel chicks born around the peak hatching date, whereas no such effect was observed for kittiwake and guillemot chicks (Figure 2; Table 2). In kittiwakes, chicks that hatched very early in the season had a higher survival, whereas in guillemots, hatching date had no apparent effect on chick survival (Figure 2; Table 2).

**Table 2:** Effect of hatching date on chick survival in three seabird species, the Antarctic petrel (a), the black‐legged kittiwake (b), and the Brünnich's guillemot (c)

Model	Np	AIC	ΔAIC
(a)			
**Hatching date + Hatching date^2^** + **Year**	**7**	**576.47**	**0.00**
(Hatching date + Hatching date^2^) × Year	13	578.09	1.62
Year	5	585.06	8.59
Hatching date + Year	6	586.64	10.17
Hatching date × Year	9	591.32	14.85
Null	2	747.77	171.30
(b)			
**Hatching date + Year**	**8**	**146.57**	**0.00**
Hatching date + Hatching date^2^ + Year	9	148.49	1.92
Hatching date × Year	13	153.27	6.70
Season	7	155.95	9.38
Null	2	213.43	66.86
(c)			
**Null**	**2**	**103.02**	**0.00**
Hatching date	3	105.00	1.98
Hatching date + Hatching date^2^	4	106.59	3.57

*Note.* Chick survival corresponds to the probability to survive to 20 (petrels) or 15 (kittiwakes and guillemots) days after hatching. Results are from linear mixed models (with a binomial residual distribution and a logit link function) that include nest identity as a random factor (see Methods). Np represents the number of identifiable parameters, AIC the Akaike's information criterion and ΔAIC the difference in AIC with the model of lowest AIC. For Brünnich's guillemot, models including year (categorical factor) did not converge and were therefore not considered; the same applied for the model (Hatching date +Hatching date^2^) × Year for black‐legged kittiwake. Sample size was 569 for petrels, 152 for kittiwakes, and 138 for guillemots (these numbers are slightly lower than numbers reported in Table 1 because a few nests with known hatching date but uncertain chick survival that were not included here). Models in bold are the ones with lowest AIC.

## 4 DISCUSSION

This study supports the hypothesis that the adaptive value of breeding synchrony depends on the predator functional response. In the Antarctic petrel, the predator was highly specialized and, as expected, petrel hatching was highly synchronous and chicks hatching close to the peak had higher survival probability. In black‐legged kittiwakes and Brünnich's guillemots, whose predators were more generalist, hatching was less synchronous and chicks hatching close to the peak did not have a higher survival probability. In kittiwakes, chicks that hatched very early in the season survived better. This higher survival for early hatched offspring has been observed in many bird species (Perrins, 1970; Verhulst & Nilsson, 2008). It is unlikely that the environmental conditions (e.g., weather, food availability) alone can explain such differences at the kittiwake colony because this effect was driven by the very first‐hatched chicks that hatch just a few days before the others (i.e., the difference in survival disappears after just a few days; see Figure 2). An alternative explanation could be that the very early breeders were high‐quality individuals (Hipfner, 1997; Verhulst, Balen, & Tinbergen, 1995; Verhulst & Nilsson, 2008). Data on adult body mass and size indicated no difference between early and late breeders (*results not shown*), but other unmeasured individual parameters, such as age or experience, could differ between early and late breeders and explain the apparent higher breeding success of early breeders (Forslund & Part, 1995).

The results indicate a significant stabilizing selection on hatching date in Antarctic petrels at Svarthamaren. This selection was not strong (selection gradient on a relative fitness scale, calculated following Janzen and Stern (1998): *γ* = ‒0.026) but was similar to the median quadratic selection via survival observed in other studies (see Kingsolver et al. 2001 for a review). The stabilizing selection on hatching date did not significantly vary among years, indicating that hatching close to the hatching peak was always associated with the highest survival during the study. This differs from a previous study in the same petrel colony. Indeed, Varpe and Tveraa (2005) reported a positive linear relationship between hatching dates and chick survival in summer 2000/01. However, as they did not test specifically for a quadratic relationship, it is unclear whether or not chick survival decreased for late breeders. If not, this would suggest that selection on hatching dates was not always stabilizing and may vary among years (Descamps et al., 2011). In summer 2000/01 at Svarthamaren, meltwater emerged around hatching and caused the death of many chicks (Varpe & Tveraa, 2005). This chick mortality, independent of predation, may have hidden the potential quadratic predation‐induced mortality.

My study focused on the first weeks after hatching, when chicks from the three species are likely extra sensitive to predation. However, the chicks may also be at risk at other times, in particular in guillemots. Indeed, Brünnich's guillemot chicks leave the colony when they are about 20 days old and only one‐third of their final mass and size (Gaston & Jones, 1998; Strøm, 2006b). This can be a critical period for their survival in some colonies (but not all, see Hatch, 1983) located away from the sea, where predation by foxes or gulls may be important when chicks land on the beach or on rocks before reaching the water (Williams, 1975). The Ossian Sarsfjellet colony is located very close to the sea and guillemot chicks apparently always reach the water when jumping (pers. obs.). In such systems, predation by gulls or foxes after jumping does not increase (Gilchrist & Gaston, 1997; Williams, 1975) and is not a function of jumping phenology (Gilchrist & Gaston, 1997). There is therefore no reason to believe that the relationship between breeding phenology and chick survival would change after jumping at the Ossian Sarsfjellet colony and that predator swamping would become an adaptive strategy to lower the predation risk.

Another potential shortcoming of this study is the confounding effect of the study area. The study systems differ in terms of predator specialization and geographical area (Arctic vs. Antarctic) and both effects are confounded. However, both systems are typical polar environments, characterized by a relatively short weather window allowing migratory birds to reproduce. I thus expect the environmental drivers behind bird phenology and breeding success in such species to be similar, both in the Arctic and in the Antarctic, and that the study systems are comparable. Additional Arctic colonies with specialized predators and/or Antarctic colonies with generalist predators would ideally be needed to tease apart the role of the region and the predator functional response. A survey of the literature provided very little information in this context as virtually no other study has linked bird phenology and success in relation to predator functional response. This study remains correlative and the observed correlations support the initial hypothesis and Ims' theoretical model (Ims, 1990b). As in all correlative studies, potential confounding factors may exist.

My results emphasize the potential importance of the timing of reproduction for individual fitness. They support the hypothesis that breeding synchrony is adaptive, increasing chick survival through a predator swamping effect, but only in predator–prey systems with specialist predators. The widely accepted view that the advantage of breeding synchronously in colonial seabirds is related to a predator swamping effect (Gochfeld,^1980^) should be considered with caution and does not necessarily apply to all species or sites. Predation is only one among many factors that can explain why breeding synchrony can be adaptive (Ims, 1990a), and several factors can potentially act simultaneously as selective forces on the same species and/or site. The relative importance of these different factors and their variation among species and sites remain to be elucidated.

## CONFLICT OF INTEREST

None declared.

## AUTHOR CONTRIBUTION

SD designed the study, wrote the manuscript, and analyzed the data.

## Data Availability

All data are available on Dryad data repository at https://doi.org/10.5061/dryad.63q3v2r.
